# Impact of neoadjuvant chemotherapy in improving operative intervention in the management of cervical cancer in low resource setting: a preliminary report

**DOI:** 10.11604/pamj.2020.36.210.24141

**Published:** 2020-07-23

**Authors:** Theophilus Ogochukwu Nwankwo, Uchenna Anthony Umeh, Uzochukwu Uzoma Aniebue, Justus Uchenna Onu, Chioma Roseline Umeh

**Affiliations:** 1 Department of Obstetrics and Gynecology, College of Medicine, University of Nigeria, Ituku-Ozalla, Enugu, Nigeria,; 2 Department of Mental Health, Faculty of Medicine, Nnamdi Azikiwe University, Awka, Nigeria,; 3 Department of Family Medicine, University of Nigeria Teaching Hospital, Ituku-Ozalla, Enugu, Nigeria

**Keywords:** Neoadjuvant, chemotherapy, cervical, cancer, radical, hysterectomy

## Abstract

There is paucity of data on interventions to improve cancer outcome in the low-resource setting. This study aims to determine the effect of Neoadjuvant Chemotherapy (NACT) in improving operative outcomes of cervical cancer. This was a longitudinal intervention study of patients diagnosed with FIGO stage IIB - IIIA cervical cancer that had NACT. Patients were re-evaluated after treatment with 4 cycles of chemotherapy for operability. McNemar test was used to determine changes in operability of the tumour. There was a significant difference in the number of patients that converted from inoperable to operable tumor post-chemotherapy. This study shows some promise for NACT for FIGO stage IIB - IIIA cancer of the cervix, especially in low-resource settings, where radiotherapy is scarce.

## Introduction

Cervical cancer is the 4^th^ commonest gynecological malignancy in women and constitutes 6.6% of all female cancers. Estimated 570,000 new cases of cervical cancer occurred in 2018 and is ranked second in incidence and mortality [1]. It is the leading cause of death in 42 countries most of which are located in sub Saharan Africa [1,2]. Many African women present with advanced or late stage of cervical cancer that are either suitable for radiotherapy or palliative care. Most African countries still lack requisite radiation equipment and where they are available; there is limited spread and access [3]. Currently there appear to be increasing involvement of chemotherapy in the management of cervical cancer [4]. A recent meta-analysis by Lapresa *et al*. [4] showed that NACT followed by surgery resulted in reduction in the risk of death by 35%, with a gain of 14% in the 5 year survival compared to radiotherapy. However, Torre *et al*. [5] did not demonstrate superiority to radiotherapy. In other words, the literature is inconsistent with regards to the usefulness of NACT in the management of 11B-111A cervical cancer [6,7].

In low resource setting, chemotherapy with surgery may be an alternative because of the paucity of radiotherapy facilities. Also, poor maintenance culture of the radiotherapy machines poses a big challenge to these patients [5]. In Nigeria, there are ten centers with capacity for radiotherapy serving over 150 million of their population. In addition, the high cost of radiotherapy coupled with non-existent health insurance coverage for the population makes the search for a viable alternative imperative. Neoadjuvant chemotherapy is used to reduce the tumor size prior to radical hysterectomy or radiotherapy [5]. Available evidence from well-designed studies suggest that neoadjuvant platinum based chemotherapy prior to definitive surgery is associated with better results than primary radiation or surgery [5,6]. Follow-up intervention studies may give further insight to which treatment option will be better for these patients. In addition, the effect of NACT on higher stages (stages 11B-111A) of cervical cancer should be explored [5-7]. With the huge burden of cervical cancer in sub-Saharan Africa and the attendant infrastructural and manpower deficit, there is need to look for other viable cost-effective treatment options to ameliorate the disease burden. In the light of the above issues, the following research question became pertinent to clinicians and researchers working on interventions to improve cervical cancer treatment in Nigeria: does neoadjuvant chemotherapy (NACT) improve the operability of stages IIB - IIIA cervical cancer in Enugu, Nigeria?

## Methods

This study was a prospective intervention study. All patients with FIGO stage 11B - 111A cervical cancer that have histological confirmation of cancer of the cervix attending gynecology clinic at University of Nigeria Teaching Hospital (UNTH) Enugu, Nigeria were recruited for the study. Study participants from the out-patient clinic and the wards were recruited when they satisfied the study criteria. A total of 33 patients with cervical cancer were seen in 30-month period of the study which was from June, 2016 to November, 2018. The 33 patients were approached and after complete description of the study, five declined to give consent due to unwillingness to participate. The remaining 28 were recruited for the study after obtaining a written informed consent. They were re-assessed and 8 patients were excluded because they had severe disease. Therefore, only 20 patients met the study criteria and were used for this preliminary report.

The treatment schedule consists of 4 cycles of intravenous cisplatin 60mg/m^2^ plus paclitaxel 60mg/m^2^ every 3 weeks. In order to avoid or minimize allergic reactions, patients were given premedication with chlorpheniramine maleate 80mg and methylprednisolone 40mg both 12 and 6 hours before each course, patients were also premedicated with ondansetron 8mg and dexamethasone 20mg, given intravenously. Antiemetics were also given for 3 days after chemotherapy. Before cisplatin administration, patients received hydration with 1,000 ml sodium chloride solution and 500ml of 5% dextrose in water. Surgery consisted of radical hysterectomy type 111 [8] and pelvic lymph node dissection. The opportunities of bilateral salpingo-oophorectomy were discussed with all patients and generally were performed in peri-menopausal women or in case of suspicious ovarian diseases; para-aortic lymphadenectomy was done. Pathological report was defined according to the TNM (Tumor Node Metastasis) classification [8]. Patients were followed up every 3 months with colposcopy and pelvic examination. The study was carried out at the University of Nigeria Teaching Hospital, Ituku-Ozalla, Enugu State, Nigeria. The Unit offer gynecological oncology treatment in Enugu and its environment. The study was a prospective observational study of eligible consenting women that presented at the gynecological clinic with histological diagnosis with FIGO stage IIB - IIIA cervical cancer that had NACT (paclitaxel and cisplatin). Patients were re-evaluated after treatment with 4 cycles of chemotherapy for operability. Data were analysed using IBM-SPSS software. McNemar test was used to determine significant changes in operability of the tumour post chemotherapy.

## Results

Mean age of participants was 57.0±9.8 years, fairly well educated (65.0%), mostly grandmultiparous women (60.0%). The commonest cervical cancer type was squamous cell (60.0%) and majority of the participants were in stage 11b at baseline. The attrition rate was 20.0%; hence 16 subjects (out of 20) were available for assessment for operability. Post chemotherapeutic reevaluation, showed a significant difference, in the number of patients that converted from inoperable to operable tumor. The McNemar test of binomial distribution for repeated measures showed a significant difference, in the number of patients that converted from inoperable tumor at baseline to operable tumor after chemotherapy. This is such that, of the 16 participants that completed the study, 15 of them were inoperable at baseline. Of this 15, 11(73.3%) became operable after chemotherapy, while 4(26.7%) remained inoperable as shown in Table 1 and Table 2 below. In brief, they were more operable tumors post-intervention (chemotherapy).

**Table 1:** socio-demographic and clinical characteristics of the participants

Variables	Frequency N=20	(%)	Mean ± S.D
Mean Age	20		57.0 ± 9.8
95%CI			52.7-61.2
**Educational Status**			
No formal	7	35.0	
Primary	10	50.0	
Secondary	2	10.0	
Tertiary	1	5.0	
**Parity**			
Primipara	0	0.0	
Multipara	8	40.0	
Grandmultipara	12	60.0	
**Cancer Type**			
Squamous cell carcinoma	12	60.0	
Adenocarcinoma	2	10.0	
Adenosquamous carcinoma	6	30.0	
**Tumor Staging at Baseline (FIGO)**			
11a	1	5.0	
11b	12	60.0	
111a	7	35.0	
**Tumor Staging after Intervention (FIGO)**			
1b	1	6.0	
11a	11	68.8	
11b	4	25.0	
Attrition Rate	4	20	
Mean Course of Chemotherapy			2.8 ± 1.0
95%CI			2.3-3.2

**Table 2:** McNemar test for the effect of chemotherapy on the tumor staging post intervention

Tumor Staging at Baseline	Tumor Staging After Chemotherapy		Statistics
	Operable	Inoperable	p=0.001^*^
Operable (n=1)	1(100.0%)	0(0.0%)	
Inoperable (n=15)	11(73.3%)	4(26.7%)	

NB: ^*^=McNemar test, Operable tumor (by FIGO Staging) = Stages 1a, 1b, and 2a, Inoperable tumor = 2b and above

## Discussion

Cancer of the cervix is the most prevalent gynecological malignancy in the developing countries [1]. Neoadjuvant chemotherapy (NACT), which can reduce the size and therefore increase the resect ability of tumors has recently evolved as an alternative treatment for locally advanced cervical cancer. The study was aimed at a treatment follow-up of 20 cases of FIGO stage 11B-111A cervical cancer patients, with a view to highlighting the changes in the operability of the tumors. The socio-demographic and clinical profile of the participants shows that they were mostly middle aged (mean age 57.0±9.8), had primary education (50.0%), and are grandmultiparous women (60.0%). The commonest cervical cancer type in this study was squamous cell (60.0%), majority of the participants were in stage 11b at baseline and the mean course of chemotherapy was 2.8 cycles. These indicate that the participants in this study were similar to the cervical cancer patients reported in previous studies [4-7]. The main highlights of the finding are that despite the efforts to ensure attendance, the attrition rate at the termination of follow-up was 20.0%; hence 16 subjects (out of 20) were available for assessment of operability. Of the 15(75.0%), that were inoperable at baseline assessment, 12(73.3%) became operable after neoadjuvant chemotherapy with cisplatin and paclitaxel. Our finding is largely consistent with the literature [6,7].

Many studies have shown that neoadjuvant chemotherapy is effective in reducing tumor size, expediting the elimination of micro metastasis, improving operability and surgical down staging [8-10]. Eddy examined the effect of neoadjuvant chemotherapy before surgery, he found 74% and 54% operability after chemotherapy for FIGO stage 11B and 111-1V, respectively [9]. In Nigeria, Khan [7] found that neoadjuvant chemotherapy followed by surgery (NACT+S) for stage 1B2 - 11B have better prognosis in terms of survival and treatment related mobility to chemoradiation. He concluded that patients tolerated NACT+Surgery better than chemoradiation. Similarly, in this study, our patients tolerated the procedure with only few patients reporting mild side effects of, diarrhea, nausea, vomiting and loss of appetite. Though, chemoradiotherapy is considered the standard treatment for FIGO Stage IIB-IVA, many researchers agree that neoadjuvant chemotherapy increases operability with minimal side effects [7,9,10]. However, with respect to survival advantage, the CCRT is superior to NACT + Surgery or radiotherapy alone [10]. In other words, in a low resource setting like Nigeria, where radiotherapy services and health insurance coverage is limited, our finding suggests some usefulness of neoadjuvant chemotherapy (NACT) and radical surgery in the management of FIGO stage 11B and 111A cervical cancer.

**Limitations:** our study is limited by the small number of participants, absence of a control group, and the use of clinical staging alone (no imaging).

## Conclusion

Neoadjuvant chemotherapy reduces tumor bulk and improves operability of stages IIB – IIIA cervical cancer disease. This study could be a winning strategy in NACT for FIGO stage IIB – IIIA cancer of the cervix, especially in low resource settings, where radiotherapy treatment is not readily available.

### What is known about this topic

- Research is inconsistent with regards to the usefulness of NACT in stages 11B-111A cervical tumor.

### What this study adds

- This study shows some usefulness of NACT in converting inoperable stage 11B-111A cervical cancer to operable tumors (stage IA-11A).
